# Evaluation of response using FDG-PET/CT and diffusion weighted MRI after radiochemotherapy of pancreatic cancer: a non-randomized, monocentric phase II clinical trial—PaCa-DD-041 (Eudra-CT 2009-011968-11)

**DOI:** 10.1007/s00066-020-01654-4

**Published:** 2020-07-07

**Authors:** Carolin Zimmermann, Marius Distler, Christina Jentsch, Sophia Blum, Gunnar Folprecht, Klaus Zöphel, Heike Polster, Esther G. C. Troost, Nasreddin Abolmaali, Jürgen Weitz, Michael Baumann, Hans-Detlev Saeger, Robert Grützmann

**Affiliations:** 1 Department of General, Thoracic and Vascular Surgery, University Hospital Carl Gustav Carus, Technische Universität Dresden, 01307 Dresden, Germany; 2 National Center for Tumor Diseases (NCT), Partner Site Dresden, German Cancer Research Center (DKFZ), Heidelberg, Germany; 3 Faculty of Medicine and University Hospital Carl Gustav Carus, Technische Universität Dresden, Dresden, Germany; 4 Association/Helmholtz-Zentrum Dresden – Rossendorf (HZDR), Dresden, Germany; 5 Department of Radiotherapy and Radiation Oncology, Faculty of Medicine and University Hospital Carl Gustav Carus, Technische Universität Dresden, Dresden, Germany; 6 Department of Radiology, Technische Universität Dresden, Dresden, Germany; 7 Medical Department I, University Hospital Carl Gustav Carus, Technische Universität Dresden, Dresden, Germany; 8 Department of Nuclear Medicine, Klinikum Chemnitz gGmbh, Chemnitz, Germany; 9 OncoRay – National Center for Radiation Research in Oncology, Faculty of Medicine and University Hospital Carl Gustav Carus, Technische Universität Dresden, Helmholtz-Zentrum Dresden – Rossendorf, Dresden, Germany; 10 German Cancer Consortium (DKTK) Partner Site Dresden, German Cancer Research Center (DKFZ), Heidelberg, Germany; 11 Department of Radiology, Municipal Hospital and Academic Teaching Hospital of the Technical University Dresden, Dresden-Friedrichstadt, Germany; 12 German Cancer Research Center (DKFZ) Heidelberg, Heidelberg, Germany; 13 Department of Surgery, University Hospital Erlangen, Erlangen, Germany

**Keywords:** Neoadjuvant radio-/chemotherapy, Downstaging, Pancreas cancer, Pancreatic adenocarcinoma, PDAC, Surgery, Imaging

## Abstract

**Background:**

Pancreatic cancer is a devastating disease with a 5-year survival rate of 20–25%. As approximately only 20% of patients diagnosed with pancreatic cancer are initially staged as resectable, it is necessary to evaluate new therapeutic approaches. Hence, neoadjuvant (radio)chemotherapy is a promising therapeutic option, especially in patients with a borderline resectable tumor. The aim of this non-randomized, monocentric, prospective, phase II clinical study was to assess the prognostic value of functional imaging techniques, i.e., [^18^F]2-fluoro-2-deoxy-d-glucose positron emission tomography/computed tomography (FDG-PET/CT) and diffusion weighted magnetic resonance imaging (DW-MRI), prior to and during neoadjuvant radiochemotherapy.

**Methods:**

Patients with histologically proven resectable, borderline resectable or unresectable non-metastatic pancreatic adenocarcinoma received induction chemotherapy followed by neoadjuvant radiochemotherapy. Patients underwent FDG-PET/CT and DW-MRI including T1- and T2-weighted sequences prior to and after neoadjuvant chemotherapy as well as following induction radiochemotherapy. The primary endpoint was the evaluation of the response as quantified by the standardized uptake value (SUV) measured with FDG-PET. Response to treatment was evaluated by FDG-PET and DW-MRI during and after the neoadjuvant course. Morphologic staging was performed using contrast-enhanced CT and contrast-enhanced MRI to decide inclusion of patients and resectability after neoadjuvant therapy. In those patients undergoing subsequent surgery, imaging findings were correlated with those of the pathologic resection specimen.

**Results:**

A total of 25 patients were enrolled in the study. The response rate measured by FDG-PET was 85% with a statistically significant decrease of the maximal SUV (SUV_max_) during therapy (*p* < 0.001). Using the mean apparent diffusion coefficient (ADC), response was not detectable with DW-MRI. After neoadjuvant treatment 16 patients underwent surgery. In 12 (48%) patients tumor resection could be performed. The median overall survival of all patients was 25 months (range: 7–38 months).

**Conclusion:**

Based on these limited patient numbers, it was possible to show that this trial design is feasible and that the neoadjuvant therapy regime was well tolerated. FDG-PET/CT may be a reliable method to evaluate response to the combined therapy. In contrast, when evaluating the response using mean ADC, DW-MRI did not show conclusive results.

**Electronic supplementary material:**

The online version of this article (10.1007/s00066-020-01654-4) contains supplementary material, which is available to authorized users.

## Introduction

Pancreatic ductal adenocarcinoma (PDAC) is associated with a poor prognosis. When detected early and treated adequately, the 1‑ and 5‑year median overall survival can be up to 20–25%. In Germany, PDAC is the third leading cancer death and the incidence corresponds to the mortality rate [1]. One of the reasons for this is that relatively few and late symptoms occur due to the organ’s localization in the retroperitoneum. Only about 20% of patients can be treated with a curatively intended resection at the time of diagnosis [2]. For patients with non-metastatic but locally advanced tumors, neoadjuvant chemo- or radiochemotherapy followed by surgery in responding patients is an option to improve outcome [3]. Ferrone et al. [4] showed a significant increase of overall survival by the neoadjuvant use of FOLFIRINOX chemotherapy.

The main potential advantages of neoadjuvant therapy include the downsizing of the tumor, increase of the resection rate, decrease of margin-positive resections, and decrease of lymph node metastases, all of which are expected to reduce the likelihood of locoregional recurrence [5]. Recent studies in patients with PDAC showed promising results regarding neoadjuvant chemotherapy or radiochemotherapy. However, as neoadjuvant treatment plus surgery increases toxicity it appears necessary to establish biomarkers for response monitoring. These may help to improve monitoring of the individual treatment of patients.

In this study on pancreatic adenocarcinoma, the value of [^18^F]2-fluoro-2-deoxy-d-glucose-positron emission tomography/computed tomography (FDG-PET/CT) and diffusion-weighted magnetic resonance imaging (DW-MRI) for the prediction of histopathological response following neoadjuvant radiochemotherapy was investigated. In previous publications, only the correlation between FDG-PET/CT findings and histopathological response had been reported for various other gastrointestinal tumors [6–8]. In particular studies on early response evaluation for cancers of the gastro-oesophageal junction have been published. Lordick et al. [6] conducted the MUNICON trial and found FDG-PET to predict early metabolic response after platinum- and fluorouracil-based induction chemotherapy. Weber et al. [8] also found FDG-PET to discriminate responders from non-responders early after cisplatin-based chemotherapy. For pancreatic cancer only a small number of studies using PET for diagnosis, staging and response can be found, thereby leaving room for this prospective study [9–11].

DW-MRI detects changes in intra- and extracellular water mobility [12, 13]. Tumors with a high cell density show a higher DW signal compared to signals arising from inflammatory processes [12]. For quantification, tissue specific properties, namely the apparent diffusion coefficient, can be calculated (ADC, [mm^2^/s]). If tumors exhibit a low ADC in initial imaging, DW-MRI may be a valuable tool for response evaluation. For pancreatic cancer, only a few reports for the use of the ADC for predicting response to chemotherapy have been published. The main reason for this is that quantitative DW-MRI is technically challenging with respect to image acquisition and analysis and to a certain extent is dependent on patient cooperation. Niwa et al. [14] reported a lower high *b*-value ADC to be predictive for early progression in patients with advanced pancreatic cancer treated with a gemcitabine-based regimen.

Based on the scarcity of data, the authors initiated a non-randomized, monocentric phase II clinical study of combined neoadjuvant chemotherapy followed by neoadjuvant radiochemotherapy with subsequent curative resection in patients with locally advanced adenocarcinoma of the pancreas. The aim was to evaluate the prognostic value of FDG-PET and DW-MRI obtained prior to and twice during neoadjuvant treatment.

## Methods and design

### Trial design and treatment

The University Cancer Center (UCC) at the Faculty of Medicine and University Hospital Carl Gustav Carus of the Technische Universität Dresden, Germany, initiated the non-randomized, monocentric, prospective phase-II-PaCa-DD-041 trial. The study was approved by the Medical Ethics Committee of the TU Dresden (EK112042010) and registered under www.clinicalregister.eu (number Eudra-CT 2009-011968-11).

The main inclusion criteria were histologically proven adenocarcinoma (by endosonography- or percutaneous image-guided biopsy) of the pancreas and medical fitness for induction chemotherapy and radiochemotherapy, possibly followed by surgery (see Table 1 for further inclusion and exclusion criteria).

**Fig. 1:**
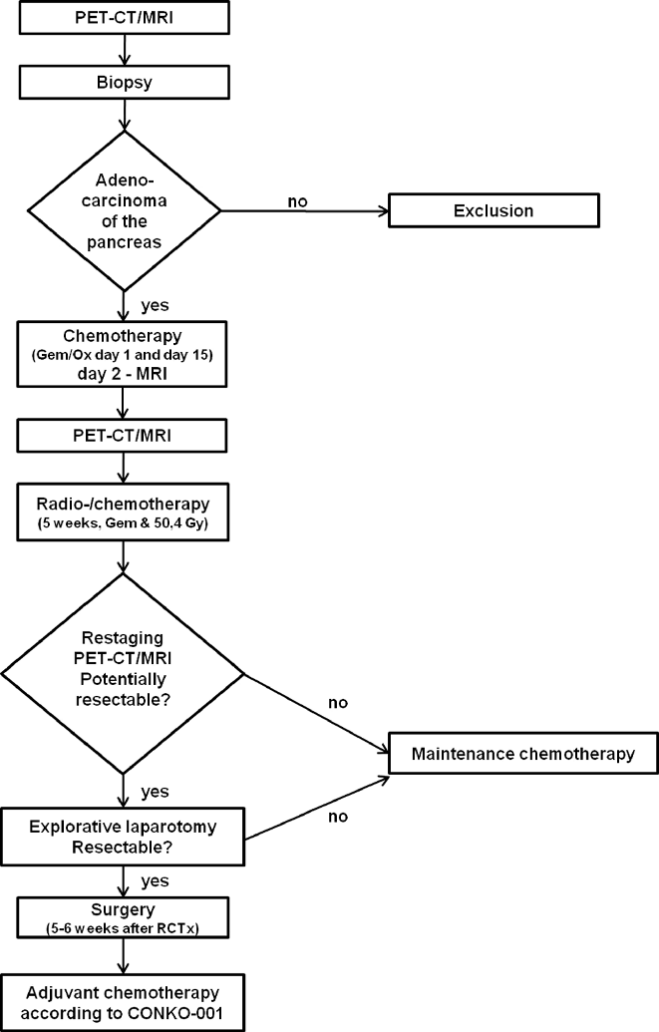
Flowchart

Eligible patients were imaged with FDG-PET/CT and morphologic magnetic resonance imaging (mMRI) and diffusion weighted magnetic resonance imaging (DW-MRI) in one session prior to induction chemotherapy, in between this and induction radiochemotherapy, and following the latter treatment. Fig. 1 depicts the study flowchart. Initial staging was based on the first FDG-PET/CT using the PET results for N and M staging and the contrast enhanced CT results for T staging. Staging for liver metastases also included contrast-enhanced mMRI. To decide on resectability of the primary tumors FDG-PET/CT and mMRI were performed after neoadjuvant therapy.

### Neoadjuvant chemotherapy

Two cycles of 1000 mg/m^2^ gemcitabine and 100 mg/m^2^ oxaliplatin were intravenously administered on days 1 and 15.

### Neoadjuvant radiochemotherapy

Radiochemotherapy was applied to a total dose of 50.4 Gy (1.8 Gy per fraction, five fractions per week) to the primary tumor, metastatic lymph nodes and elective lymph node stations, and an additional boost of 5.4 Gy to the primary tumor and affected lymph nodes. Radiation treatment planning was based on a four-dimensional CT taking into account respiratory motion. On the CT, the gross tumor volume (GTV) was contoured in all phases and the sum of these GTVs resulted in the internal target volume (ITV) for the boost. This ITV was isotropically expanded by 5 mm (corrected for anatomical boundaries) to reach the clinical target volume (CTV), and the planning target volumes (PTV) was calculated as CTV with a margin of 10 mm. Organs at risk contoured included the right and left kidney, liver, small bowel and spinal cord. Tc99m renal scintigraphy was performed in all patients to assess adequate bilateral kidney function for radiation treatment. Concomitant gemcitabine was intravenously administered at days 1, 8, 15, 22 and 29 at a dose of 300 mg/m^2^.

### Surgery

Patients with non-metastatic resectable or borderline resectable disease underwent surgery within 6 weeks after neoadjuvant treatment. Contact with, or infiltration of, the portal vein was not a criterion for unresectability. An unresectable tumor was defined as one which surrounded the common hepatic artery or adhered to the right or left hepatic artery, superior mesenteric artery or the coeliac trunc [15]. The decision for resectability was based on the results of the contrast-enhanced CT as part of the FDG-PET/CT that was acquired after radiochemotherapy.

### Adjuvant and palliative chemotherapy

Surgery was followed by adjuvant chemotherapy according to CONKO-001 [16]. Patients with metastatic or unresectable disease at the time of re-staging following neoadjuvant therapy received palliative chemotherapy with gemcitabine and oxaliplatin or other treatment regimes such as FOLFIRINOX [17].

### FDG-PET/CT and MRI

FDG-PET/CT and MRI images were obtained before treatment, after the two courses of neoadjuvant chemotherapy (day 21) and after neoadjuvant radiochemotherapy (10 weeks after radiochemotherapy). Additionally, MRI was obtained on day 2 of chemotherapy for very early response assessment.

PET imaging was performed with FDG to determine metabolic activity and response of the tumor. For this, patients received 5 MBq FDG/kg bodyweight after having fasted for 6 h prior to the PET examination. Along with the FDG-PET scan, which was obtained approximately 60 min after tracer administration, a CT with intravenous contrast agent was obtained, also serving for attenuation correction purposes. All patients were examined with a standardized protocol in supine position on a Biograph 16 scanner (Siemens Medical Solutions Inc., Knoxville, TN). For FDG-PET analysis, the maximum standardized uptake value (SUV_max_) was determined. Metabolic response was defined as a decrease of the SUV_max_ of ≥30% compared to the pretreatment FDG-PET SUV_max_.

All patients were examined with a standardized protocol on a 1.5‑T scanner (Siemens Somatom Avanto, Siemens Healthcare, Forchheim, Germany). To obtain morphological as well as functional images, the following sequences were included: T2-weighted (T2-w) coronal and transversal images, T1-weighted (T1-w) transversal images, coronal and transversal T1‑w images after administration of 0.2 ml/kg Gadobutrol (Gadovist®, 1 mmol/l solution, Bayer Pharma AG, Leverkusen, Germany), transversal diffusion-weighted images with b‑values of 0, 50, 100, 150, 400, 600 and 800 mm^2^/s. Using custom-made dedicated software (Geisterr), tumor volumes were manually contoured using T1‑w and T2‑w imaging. These contours were copied to the DW images of all b‑values registered to the morphologic sequences by the same software. From the latter, quantitative transversal ADC maps were calculated using the b‑values from 400 to 800 mm^2^/s. Tumor size was measured in contrast-enhanced T1‑w images according to RECIST 1.1 guidelines [18]. Morphologic response was defined as a decrease of tumor size by ≥30% compared to the pretreatment size and functional response was defined as an increase of the ADC values by ≥30% compared to pretreatment ADC.

### Endpoints

The main endpoint of the study was the evaluation of the response to neoadjuvant treatment by measuring the FDG-PET SUV_max_. Secondary endpoints included an evaluation of the response using DW-MRI, assessment of toxicity (according to the Common Terminology Criteria for Adverse Events v4.0 [CTCAE]), recurrence-free survival, overall survival, toxicity, R0 resection rate, postoperative complications according to the Clavien-Dindo classification [19] and perioperative mortality and morbidity, as well as postoperative mortality (during hospitalization or within 30 days after surgery). The maximum follow-up was 48 months. The results of the imaging parameters are presented in this publication.

### Biometry

In this study a response rate of 15% was expected. To calculate the response rate, a 95% confidence interval with the lower confidence bound of 7.5% was defined. Therefore, a study population of 62 analyzable patients was needed. Since an expected exclusion rate of 10% due to another result in the definite pathology, a final recruiting rate of 70 patients was calculated. The secondary endpoints were analyzed by exploratory data analysis. For continuous variables mean, median and confidence interval were defined. Categorical variables were analyzed by absolute and relative frequency. Overall survival was estimated using the Kaplan-Meier method. Survival was defined as the time from informed consent to death. The statistical analysis was performed by SPSS, version 17, and by SAS for windows, version 9.2.

## Results

A total of 50 patients suspected of having PDAC were screened between 06/2011 and 12/2015. The median patient age was 68 years, 14 (61%) were female, the Karnofsky Index was 90 and in 83% (19 patients) the tumors were located in the head of the pancreas. Of the screened patients, 13 had metastatic disease during the staging procedures, four had other pathological findings, one had a second synchronous tumor, in four patients adenocarcinoma could not be pathologically confirmed, and four were excluded for other reasons. Finally, 25 patients were enrolled in the study. Of these, two patients were excluded due to early development of metastatic disease and cardiac decompensation during the first cycle of chemotherapy, leaving 23 patients available for analysis. Since the study was poorly recruiting, it was terminated prior to accrual of the calculated number of patients. All enrolled patients received their therapy according to protocol.

### Staging and restaging

Initial staging by CT and MRI categorized 13% of the primary tumors as resectable, 30% as borderline resectable and 56% as unresectable. After completion of the neoadjuvant treatment restaging was performed according to the RECIST criteria [20]. Three patients (13%) showed complete remission, another three showed partial remission and the remaining patients showed stable disease based on CT using RECIST criteria.

### Surgery

After completion of the neoadjuvant therapy, surgery was performed in 16 (69%) of the enrolled patients, 12 of which underwent R0 resection (Table 4). Depending on the localization of the tumor the operation was performed as pylorus-preserving pancreatico-duodenectomy (PPPD) in seven patients, as Kausch-Whipple in one patient, as left resection in two patients and as total pancreatectomy in two patients. Resection of the portal vein was necessary in 10 patients and in four patients concomitant arterial resection was performed. This and the histopathologic result of an ypT3 stage in 82% showed that most of the patients initially had a locally advanced tumor stage.

### FDG-PET imaging

**Fig. 2:**
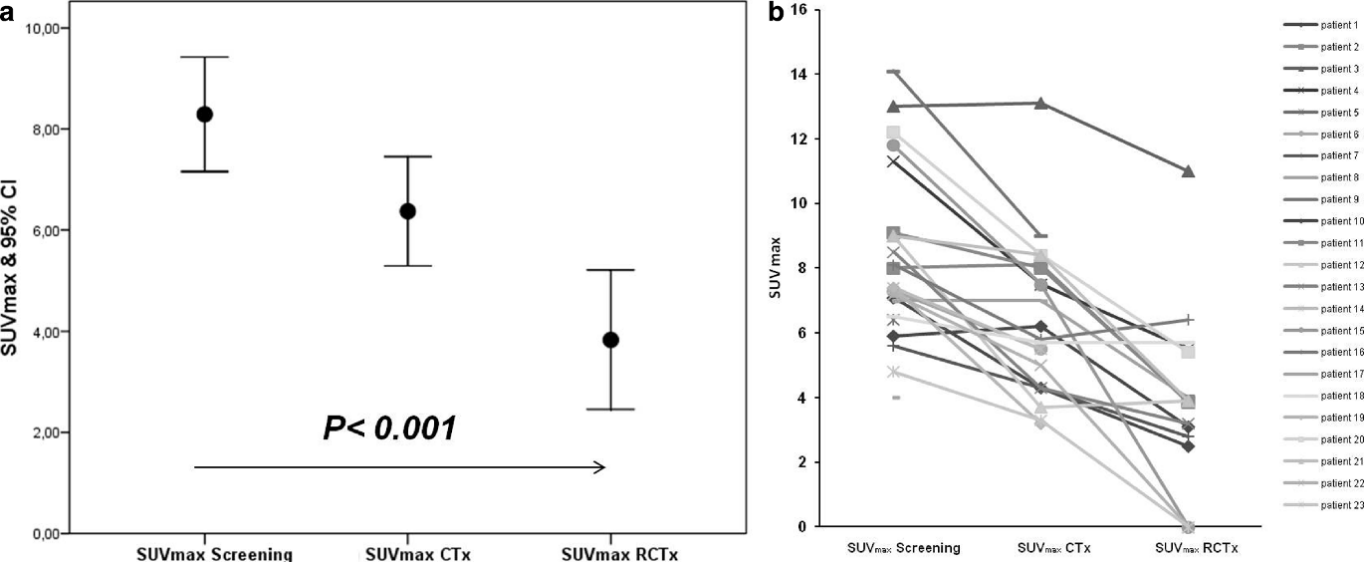
Maximum standardized uptake value (*SUV*_*max*_) for [^18^F]2-fluoro-2-deoxy-d-glucose-positron emission tomography/computed tomography of pancreatic adenocarcinoma before neoadjuvant treatment, after two cycles of chemotherapy and after completion of neoadjuvant radiochemotherapy, *error bar* and *line diagram* of the SUV_max_. **a** Statistically significant decrease of metabolic activity in the cohort of patients over the neoadjuvant treatment; screening; mean ± standard deviation (SD): 8.29 ± 2.62 (95% confidence interval [CI]: 7.15–9.42); after completion of chemotherapy (*CTx*), mean ± SD: 6.37 ± 2.37 (95% CI: 5.29–7.45); after completion of radiochemotherapy (*RCTx*); mean ± SD: 3.83 ± 2.67 (95% CI: 2.46–5.21) (*p* = 0.0001*, *one-way analysis of variance). **b** SUV_max_ in individual patients, overall 15 of 18 patients showed a metabolic response

FDG-PET identified 15 patients (85%) as metabolic responders. Only three tumors (15%) showed a decrease of the SUV_max_ of less than 30% and were defined as non-responders. The median SUV_max_ values for the patient cohort decreased statistically significantly when comparing before and after completion of neoadjuvant therapy (SUV_max_ = 8.29 and 3.83, respectively; *p* < 0.001; Fig. 2). Five patients (41.7%) showed both metabolic and histopathological responses defined by Becker et al. [21]. Another five patients (41.7%) had only a moderate histopathological response despite a significant response in FDG-PET. Two patients (16.7%) were identified as histopathological and morphological non-responders.

### Diffusion weighted MR imaging

**Fig. 3:**
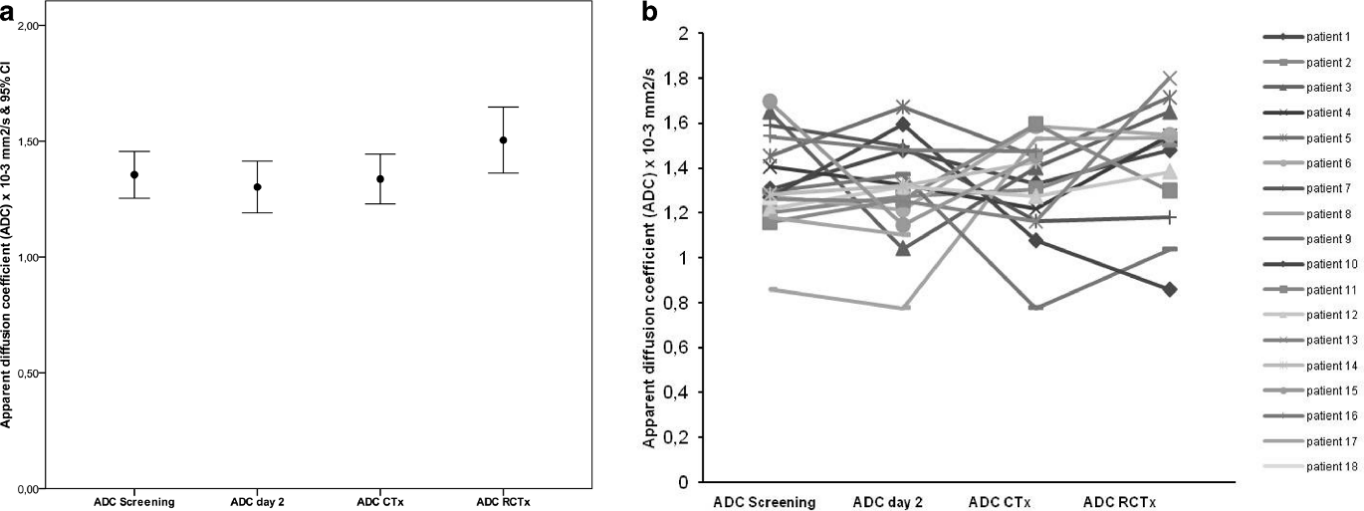
Apparent diffusion coefficient (*ADC*) determined by diffusion-weighted magnetic resonance imaging before neoadjuvant treatment of pancreatic adenocarcinoma, after 2 days of the first cycle of chemotherapy, after two cycles of chemotherapy and after completion of neoadjuvant radiochemotherapy, *error bar* and *individual line diagram* of the ADC. **a** ADC at screening; mean ± standard deviation (SD): 1.32 ± 0.20 (95% confidence interval [CI]: 1.23–1.43); ADC at day 2 after first application of chemotherapy (gem/ox); mean ± SD: 1.30 ± 0.22 (95% CI: 1.19–1.41); ADC after completion of chemotherapy (*CTx*); mean ± SD: 1.32 ± 0.21 (95% CI: 1.21–1.44); ADC after completion of radiochemotherapy (*RCTx*); mean ± SD:1.43 ± 0.27 (95% CI: 1.26–1.59); *p* = 0.468 (one-way analysis of variance). **b** ADC in individual patients with highly variable response during the course of the study. The mean ADC did not show a distinct change in diffusion restriction

During the neoadjuvant therapy as administered within the PaCa-DD-041 trial, there were no statistically significant changes in ADC values (mean ADC values: 1.32 prior to treatment; 1.30 and 1.32 prior to and after induction chemotherapy; 1.43 ± after completion of neoadjuvant radiochemotherapy; *p* = 0.468; Fig. 3). The reduction in tumor size according to RECIST 1.1 criteria after therapy was also not statistically significant (*p* = 0.057).

### Safety of the neoadjuvant therapy

The grade 2–4 toxicities are listed in Table 5. Grade 5 toxicities did not occur during this clinical trial. Most of the toxicities were hematological changes, such as neutropenia and leucopenia, and were related to chemotherapy.

### Survival

**Fig. 4:**
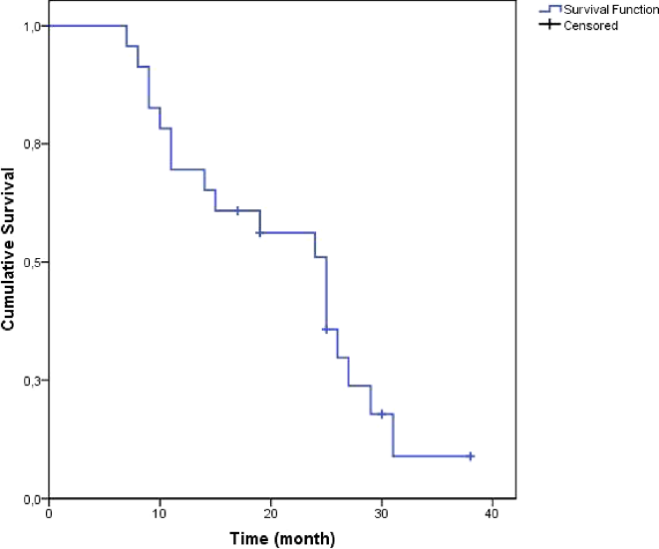
Kaplan-Meier overall survival (*N* = 23), median overall survival: 25 months (95% confidence interval: 18.9–31)

Fig. 4 shows the Kaplan-Meier overall survival curve of the entire study cohort. Of the enrolled patients, 19 had died and four patients were alive (censored) at the time of analysis. Median overall survival was 25 months and 1‑year survival was 70%.

## Discussion

In routine staging for PDAC, FDG-PET plays only a minor role due to its low specificity and sensitivity of 61–94% and 85–97%, respectively [22, 23]. The main advantage of FDG-PET is its sensitive detection of distant metastases; furthermore, FDG-PET is a good method for the evaluation of metabolic response. This has been shown in numerous other tumor entities [24, 25]. In this trial using the decrease of SUV_max_, the majority of patients were detected as responders and only a minority as non-responders. However, a clear correlation between responders in FDG-PET and histopathological response could not be found. Based on these findings, it is not possible to predict histopathological response on the basis of metabolic FDG-PET response. A number of other studies had established a correlation between the metabolic and histopathological response [6–8].

It can be concluded that, by using FDG-PET/CT, the selection of patients with non-responding or progressive disease is possible. Thus, patients can be offered alternative therapeutic approaches, such as intensified neoadjuvant chemotherapy or a palliative treatment regimen early on.

Due to a distinct desmoplastic reaction induced by neoadjuvant radiotherapy no significant decrease in tumor size on the CT scan is expected [4, 26]. Therefore, the response can be poorly estimated and lead to misinterpretation. As expected, the authors were not able to show size changes of the tumors after neoadjuvant radiotherapy as measured by morphologic MRI. Due to this, as well as changes in tumor cellularity, the use of DW-MRI is increasingly considered [27]. Based on the data from this trial, however, the evaluation of the response with DW-MRI was not possible, with the ADC values showing no significant correlation. This could stem from fibrosis induced by radiochemotherapy. So far, only small cohort studies have been conducted and, for the future, studies with larger cohorts are required [28]. One major limitation of quantitative DW-MRI of the pancreas is its high sensitivity to motion artifacts, both to movement of the organ and to phase encoding artifacts due to bowel motion. When performing DW-MRI, high costs need to be taken into account as major downsides of this imaging modality. Nevertheless, DW-MRI seems to be an interesting non-invasive option for short-term follow-up during oncologic therapy of various primary tumors [29–31]. The authors believe that further developments in this area will increase the diagnostic performance of DW-MRI.

Regarding the neoadjuvant therapy in the PaCa-DD-041 trial, the feasibility of the induction chemotherapy with gemcitabine and oxaliplatin, followed by radiochemotherapy with gemcitabine, was shown. Generally, the therapy was well tolerated and no grade 5 toxicities were observed. As determined using the RECIST criteria, a response (partial and complete) rate of 26% was recorded. In all, 61% of the study population showed stable disease. The high rates of stable disease can be explained by the moderate cytotoxicity of gemcitabine and oxaliplatin. By using more effective regimes like FOLFIRINOX, response rates can possibly be increased [17].

In summary, the PaCa-DD-041 trial was terminated early due to low accrual. A significant metabolic response based on FDG-PET was found in 50% of patients; however, this did not correlate with pathologic response following neoadjuvant treatment. Nevertheless, by using FDG-PET, monitoring of neoadjuvant therapy for trend prediction and to avoid ineffective and potentially harmful treatment is possible. The authors did not find a potential role for DW-MRI in early response evaluation. Further research needs to show whether FDG-PET response evaluation saves patients from undergoing ineffective, potentially toxic treatment.

## Caption Electronic Supplementary Material

Figure 1: Consort Flow Diagram of PaCa-DD-041; Figure 2: 72-year old female patient, adenocarcinoma of the pancreas head, initially not resectable (surrounding of the superior mesenteric artery and contacting of confluence), SUVmax 8.3; Figure 3: PET-CT scan after completion of the radio-/chemotherapy, SUVmax decreases about 54%, SUVmax 3.85, patient underwent operation: PPPD with partial resection of the portal vein, histology: ypT3, ypN0, M0, L0, R0, less than 10% vital tumor cells; Figure 4: contrast-enhanced fat saturated T1-weighted image, white arrow: 36.3 mm tumor of the pancreas head; Figure 5: a) ADC map b) DW image with a b-value of 800 sec/mm2, in both pictures the tumor is seen (black and white arrow)
Table 1: Inclusion and exclusion criteria; Table 2: Baseline patients demographics, treatment parameters (*N* = 23); Table 4: Intra- and postoperative criteria; Table 5: Toxicities of the neoadjuvant chemotherapy and radiochemotherapy rated by the Common Terminology Criteria for Adverse Events v4.0 (CTCAE), the values given are number (percentages)

§§

## Abbreviations

ADC: Apparent diffusion coefficient
CA 19‑9: Cancer antigen 19‑9
CEA: Carcinoembryonic antigen
CI: Confidence interval
CT: Computed tomography
DW: Diffusion weighted
FDG: [^18^F]2-fluoro-2-deoxy-d-glucose
Gy: Gray
MRI: Magnetic resonance imaging
OS: Overall survival
PDAC: Pancreatic ductal adenocarcinoma
PET-CT: Positron emission tomography–computed tomography
PPPD: Pylorus-preserving pancreatico-duodenectomy
SUV_max_: Maximum standardized uptake value
