# Supplementary material for: Evaluation of response using FDG-PET/CT and diffusion weighted MRI after radiochemotherapy of pancreatic cancer: a non-randomized, monocentric phase II clinical trial—PaCa-DD-041 (Eudra-CT 2009-011968-11)
Source: Strahlenther Onkol. 2020 Jul 7;197(1):19–26. doi: 10.1007/s00066-020-01654-4 (PMC7801319; doi:10.1007/s00066-020-01654-4)
Supplement: Supplementary file 1 — Figure 1: Consort Flow Diagram of PaCa-DD-041; Figure 2: 72-year old female patient, adenocarcinoma of the pancreas head, initially not resectable (surrounding of the superior mesenteric artery and contacting of confluence), SUVmax 8.3; Figure 3: PET-CT scan after completion of the radio-/chemotherapy, SUVmax decreases about 54%, SUVmax 3.85, patient underwent operation: PPPD with partial resection of the portal vein, histology: ypT3, ypN0, M0, L0, R0, less than 10% vital tumor cells; Figure 4: contrast-enhanced fat saturated T1-weighted image, white arrow: 36.3 mm tumor of the pancreas head; Figure 5: a) ADC map b) DW image with a b-value of 800 sec/mm2, in both pictures the tumor is seen (black and white arrow) Table 1: Inclusion and exclusion criteria; Table 2: Baseline patients demographics, treatment parameters (N = 23); Table 4: Intra- and postoperative criteria; Table 5: Toxicities of the neoadjuvant chemotherapy and radiochemotherapy rated by the Common Terminology Criteria for Adverse Events v4.0 (CTCAE), the values given are number (percentages) [file 66_2020_1654_MOESM1_ESM.docx]

**Supplementary Figures:**

## Enrollment

Allocated to intervention (n= 25)

♦ Received allocated intervention (n= 23)

♦ Did not receive allocated intervention

(metastatic disease and cardial

decompensation) (n= 2)

Lost to follow-up (n= 0)

Analyzed (n= 23)

Included (n= 25)

Excluded (n= 25)

♦  metastatic disease (n= 13)

♦ other pathological finding (n= 4)

♦  pathological confirmation failed (n= 4 )

♦  Other reasons (n= 5)

Assessed for eligibility (n= 50)

## Allocation

## Follow-Up

## Analysis

**Figure 1:** Consort Flow Diagram of PaCa-DD-041

**Figure 2:** 72-year old female patient, adenocarcinoma of the pancreas head, initially not resectable (surrounding of the superior mesenteric artery and contacting of confluence), SUV_max_ 8.3

**
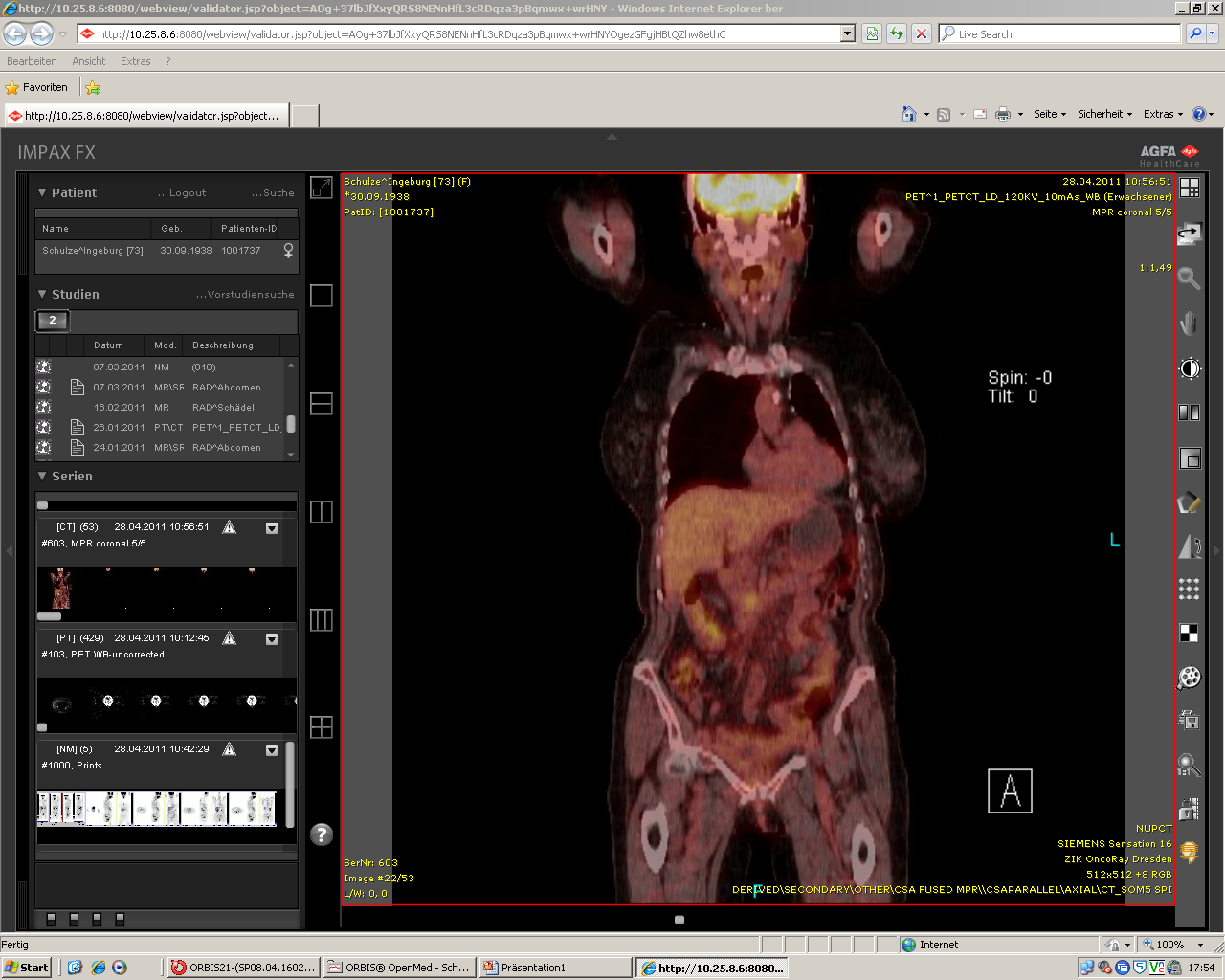

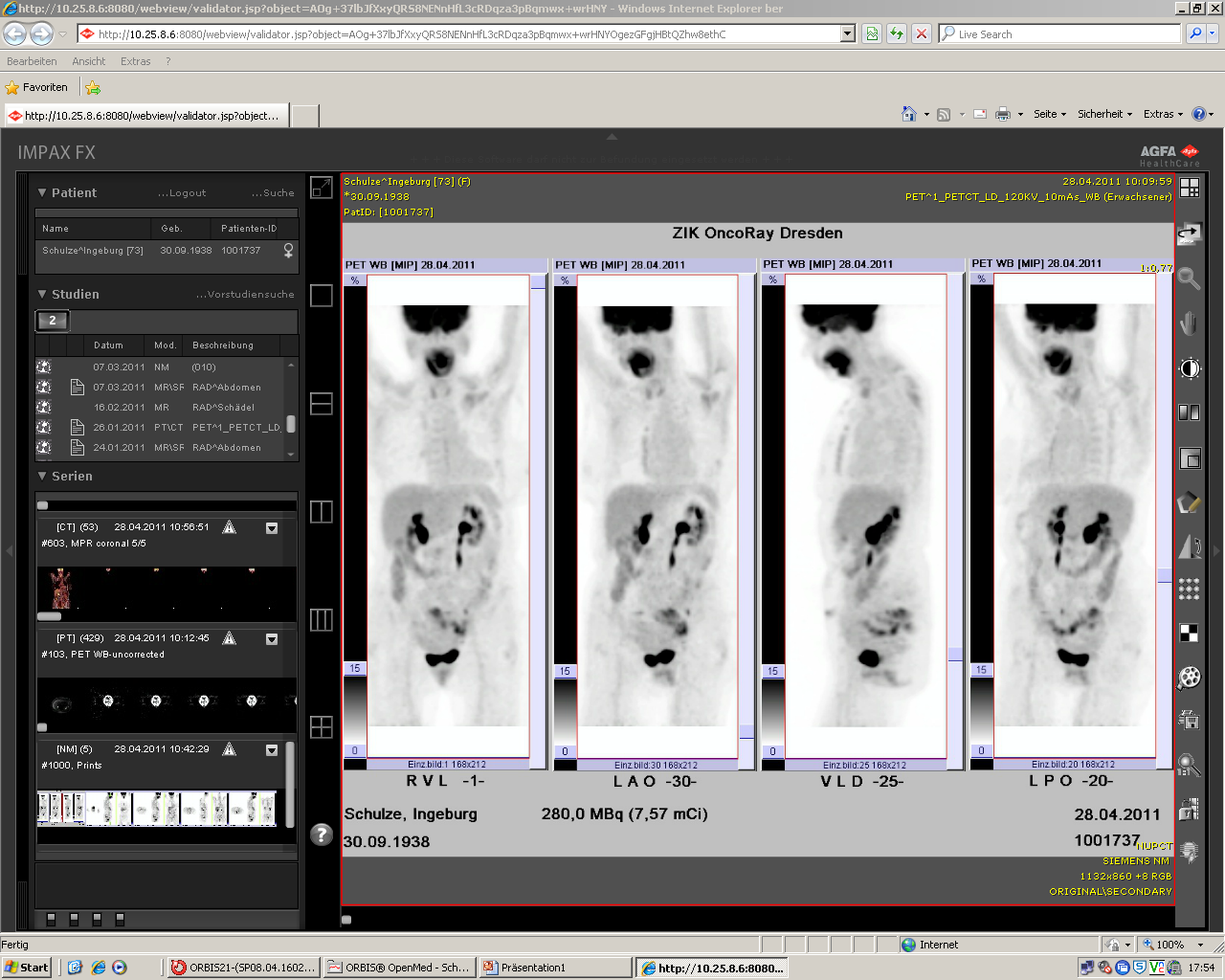
**

**Figure 3:** PET-CT scan after completion of the radio-/chemotherapy, SUV_max_ decreases about 54%, SUV_max_ 3.85_,_ patient underwent operation: PPPD with partial resection of the portal vein, histology: ypT3, ypN0, M0, L0, R0, less than 10% vital tumor cells

**
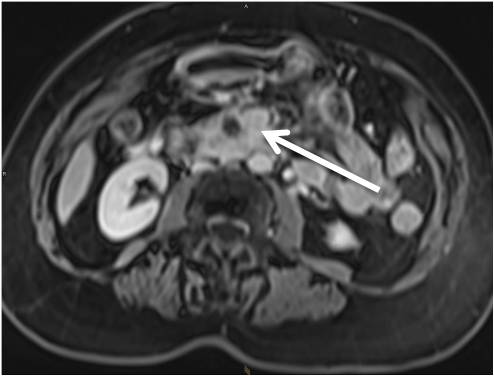
**

**
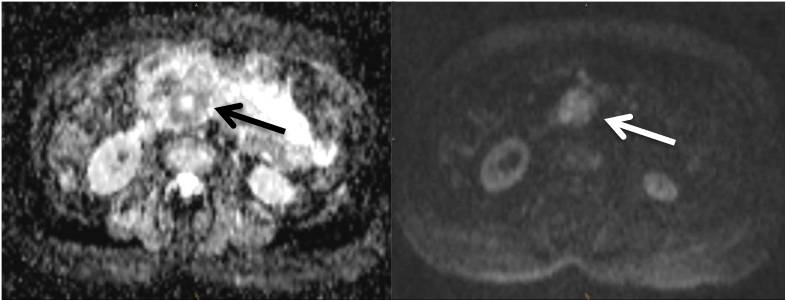
Figure 4:** contrast-enhanced fat saturated T1-weighted image, white arrow: 36.3 mm tumor of the pancreas head

**Figure 5:** a) ADC map b) DW image with a b-value of 800 sec/mm^2^, in both pictures the tumor is seen (black and white arrow)

a

b
