# Supplementary material for: Evaluation of response using FDG-PET/CT and diffusion weighted MRI after radiochemotherapy of pancreatic cancer: a non-randomized, monocentric phase II clinical trial—PaCa-DD-041 (Eudra-CT 2009-011968-11)
Source: Strahlenther Onkol. 2020 Jul 7;197(1):19–26. doi: 10.1007/s00066-020-01654-4 (PMC7801319; doi:10.1007/s00066-020-01654-4)
Supplement: Supplementary file 2 — §§ [file 66_2020_1654_MOESM2_ESM.docx]

**Supplementary Tables:**

**Table 1:** Inclusion and exclusion criteria

| Inclusion criteria |
| --- |
| - Histologically-proven adenocarcinoma of the pancreas |
| - Radiographically detectable tumor - Patients medically fit for chemotherapy and radiochemotherapy |
| - Men and Women aged 18 years or more |
| - Karnofsky performance status ≥ 80% |
| - Written informed consent |
| Exclusion criteria |
| - Ampullary carcinoma |
| - Other pancreatic carcinomas such as neuroendocrine tumors |
| - Recurrent disease |
| - Metastases to distant organs (liver, peritoneum, lung, others) |
| - Earlier or synchronous tumors |
| - Cirrhosis of the liver with thrombocytes < 100.000 Gpt/l |
| - Severe cardiopulmonary disease |
| - HIV infection |
| - Immunosuppression |
| - Severe wounds, ulcers or fractures |
| - Suppression of the spinal cord before starting treatment with oxaliplatin |
| - Peripheral sensory neuropathy |
| - Patients with ferromagnetic implants |
| - Addiction to drugs or other medical, familial, sociological or psychological conditions which could potentially hamper compliance with the study protocol or follow-up |
| - Pregnant or nursing women |
| - Participation in other clinical trials during the last four weeks before allocation to this trial |

**Table 2:** Baseline patients demographics, treatment parameters (N= 23)

| Demographic characteristics (N= 23) | N patients (%) |
| --- | --- |
| Gender  male  female | 9 (39)  14 (61) |
| Age (Median) (range)  male  female | 68 (49 – 75)  66  69 |
| Karnofsky-Index | 90 |
| Primary localization of the tumor  head  body/tail | 19 (83)  4 (17) |
| Histology: adenocarcinoma | 23 (100) |

**Table 4:** Intra- and postoperative criteria

| Surgery and histopathology (N= 23) | N patients (%) |
| --- | --- |
| Exploration | 16 (69) |
| Resectable after completion of radio-/chemotherapy | 12 (52) |
| Histology: adenocarcinoma (N=12) | 12 (100) |
| T-Stage  ypT0  ypT1  ypT3 | 1 (9)  1 (9)  10 (82) |
| Lymph nodes (N=12)  ypN0  ypN1 | 11 (92)  1 (8) |
| Resection status (N=12)  R0  R1  Rx | 9 (75)  0  3 (25) |
| CRM + | 1 (8) |
| Lymph-vascular/perineural invasion  Lymph-vascular (N= 12)  Perineural (N=10) | 1 (8)  8 (80) |
| histopathologic response  1 (0 – 25 %, minor)  2 (26 – 50 %, moderate)  3 (51 – 75 %, major) | 3 (25)  4 (33)  4 (33) |
| 4 (> 75 %, complete) | 1 (8) |
| Irresectable (N 16)  Liver metastases  peritoneal carcinomatosis  Infiltration of the root of mesentery | 4 (25)  1 (6)  1 (6)  2 (12,5) |

| **Toxicity (N= 23)** | **Grade 2** | **Grade 3** | **Grade 4** |
| --- | --- | --- | --- |
| *Hematological toxicity*  Anemia  Neutropenia  Leukopenia  Thrombocytopenia  Pancytopenia  Hyperglycemia | 5 (6)  10 (12)  19 (23)  4 (5) | 3 (3,6)  5 (6)  1 (1)  1 (1)  1 (1) | 1 (1)  1 (1) |
| *Gastrointestinal* *symptoms*  Abdominal pain  Nausea/vomiting  Infection (cholangitis /  perforated cholecystitis /  sigmoid diverticulitis)  Elevated liver enzymes | 2 (2,5)  6 (7)  3 (3,6) | 1 (1)  3 (3,6)  1 (1) | 2 (2,5) |
| *Constitutional symptoms*  Fatigue  Weight loss  Fever | 5 (6)  1 (1) | 1 (1) |  |
| *Other toxicities*  Neuropathy  Cardiac decompensation  Hypertension  Pulmonary embolism  Portal vein thrombosis  Sepsis  Circulatory dysregulation  Injection site reaction | 1 (1)  1 (1)  1 (1) | 1 (1)  1 (1)  1 (1) | 1 (1) |

**Table 5:** Toxicities of the neoadjuvant chemotherapy and radiochemotherapy rated by the Common Terminology Criteria for Adverse Events v4.0 (CTCAE), the values given are number (percentages)
